# The Impact of COVID-19 Pandemic on Practice Patterns and Psychological Status of Ophthalmologists in Turkey

**DOI:** 10.7759/cureus.16614

**Published:** 2021-07-25

**Authors:** Ceren Durmaz Engin, Basak Senel Kara, Taylan Ozturk, Omer Faruk Dadas

**Affiliations:** 1 Ophthalmology, Karadeniz Ereğli State Hospital, Zonguldak, TUR; 2 Psychiatry, Karadeniz Ereğli State Hospital, Zonguldak, TUR; 3 Ophthalmology, Dokuz Eylül University Faculty of Medicine, Izmir, TUR; 4 Biostatistics and Medical Informatics, Ege University Faculty of Medicine, Izmir, TUR

**Keywords:** anxiety, coronavirus, depression, insomnia, ophthalmologists, stress, covid-19

## Abstract

Aim: To investigate the changes in ophthalmologists' working conditions and mental health status in Turkey during the first wave of the COVID-19 outbreak and reveal the relevant individual and workplace-related factors.

Methods: This cross-sectional, nationwide, the survey-based study collected data between June and September 2020. Demographic characteristics, working conditions, precautionary measures in the workplace, and participants' Depression Anxiety Stress Scale (DASS-21) and Insomnia Severity Index (ISI) ratings were investigated.

Results: This study included 360 actively working ophthalmologists. While 64% of them worked in the pandemic hospitals, 44% were actively involved in COVID-related departments. Among those, 56 (35%) declared that they had all personal protective equipment in sufficient quantity in their COVID department. Despite the restrictions, 32% reported continuing to see 25 to 50 patients per day in ophthalmology clinics, with the most common complaint being the ocular "itching and burning" sensation. 53% stated that they did not perform any surgeries. Symptoms of depression, anxiety, stress and insomnia were present in 65%, 56.9%, and 43% and 46.9% of participants, respectively. All DASS-21 subscales and ISI scores were found to be significantly higher during the pandemic. Female gender, older age, and lower satisfaction levels of hygiene conditions in COVID clinics were independent predictors of higher DASS-21 subscale scores in multivariate analysis. Being a resident was a major predictor of depression. Ophthalmologists working in a pandemic hospital were more likely to experience insomnia.

Conclusion: Ophthalmologists have actively worked in COVID departments during the pandemic. Increased psychological distress among ophthalmologists compared to the pre-pandemic period is caused by personal factors and many determinants related to the workplace and practice patterns. Therefore, decreasing the transmission risk by creating a protective workplace and developing psychological support policies should be considered to minimize adverse psychological effects.

## Introduction

In late December 2019, the outbreak of a new coronavirus (COVID-19) was reported in Wuhan, China. Despite many preventive measures, Turkey has been ranked among the top 10 countries with the highest cases of COVID-19 worldwide within the first 30 days of the epidemic. In order to deal with a high number of cases, as of 20th of March, most of the state and private hospitals have been declared as pandemic hospitals by the Ministry of Health (MOH), and hospitals rapidly reconfigured clinical spaces and restructured clinical teams. Measures such as restriction on the number of patients evaluated in the outpatient clinics, postponement of elective surgeries, and alternating work were taken to prevent the possible accumulation in the healthcare system [1]. Additionally, many healthcare workers (HCW) were redeployed to COVID-19 clinics outside of their clinical expertise. As of April 2021, nearly 200.000 HCW were infected with COVID-19, and 362 HCW died, including 122 doctors in Turkey [2].

Insufficient supply of personal protective equipment (PPE), long working hours, high risk of getting infected and spreading an infection to family members have negatively affected the mental health status (MHS) of HCW during the COVID-19 pandemic. 24-hour shifts have caused disturbances in their sleep patterns [3,4]. These mental health problems affect not only the attention and decision-making skills of medical workers which could hinder the fight against COVID-19 but also have a lasting impact on their overall well-being. In addition to this, little was known about the new coronavirus during the first wave of the pandemic, including its lethality, transmission rate, protective measures, and how to best care for these patients. This lack of knowledge increased the mental distress experienced by HCW [5].

The ophthalmologic examination requires close contact with the patients, increasing the risk of virus spread through direct contact since SARS-CoV-2 exists in tears and conjunctival secretions [6]. High transmission risk, along with concerns of unfamiliar clinical roles in dealing with COVID‑19, may negatively affect the MHS of ophthalmologists. In this study, 360 ophthalmologists in Turkey were surveyed to determine working conditions during the first wave of pandemic and reveal the presence and severity of depression, anxiety, stress-related symptoms and insomnia. The prevalence of these symptoms during the pandemic was compared with the pre-pandemic period. Personal and work-related factors contributing to mental health problems were investigated. Workplace conditions that can be modified by the institution, such as the adequacy of PPE and hygiene conditions, were particularly questioned. Thus, the study findings were intended to provide a scientific basis for policymakers to develop psychological support policies and create a protective work environment.

This study was presented as a poster at the annual meeting of the Turkish Ophthalmological Association, Online in November 2020.

## Materials and methods

Study Design and Participants

A web-based questionnaire was applied to 360 actively working ophthalmologists who volunteered for the study between June and September 2020. The questionnaire was prepared on Google Forms and sent via personal e-mails, and circulated on different online platforms. A total of 385 respondents completed the questionnaire. To ensure the quality of the questionnaire, participants over the age of 65, questionnaires involving the same answers given in a row, invalid content and incomplete data were excluded. Finally, a total of 360 valid questionnaires were included in the study. The sample size required to achieve a 95% confidence interval was calculated as 357 based on the total number of ophthalmologists working in Turkey.

The study was approved by both the Turkish MOH and the Local Ethics Committee and conducted following the principles of the Declaration of Helsinki. The study objectives were explained to participants, and electronic informed consent was obtained from each participant.

Questionnaire

The questionnaire included questions on sociodemographic characteristics, including gender, age, marital status, health status, job title, practice patterns, workplace conditions and anxiety-depressive-stress and insomnia symptomology. The extent of training given by the institution to the physician about post-COVID regulations and sufficiency of PPE indicated in MOH guidelines were evaluated with yes/no type questions. A participant was given 1 point for each item marked as yes, and the total scores were determined as the "Degree of training" and the "Sufficiency of PPE", respectively. Participants were asked to evaluate the hygiene conditions of both COVID and ophthalmology clinics by a Likert type scale ranging from quite inadequate (1 point) to quite adequate (5 points). Each participant's total score was accepted as "The satisfaction level of physician of hygiene conditions" and used in statistical analysis. The number of patients examined and the number of nasopharyngeal swabs (NPS) taken daily was questioned. Finally, the participants were asked how much the factors such as personal contamination, transmission to family members, social isolation, stigmatization, fighting an illness whose exact treatment was unknown, and practising in a non-disciplinary field affect their level of distress with a Likert type scale ranging from never (1 point) to very severe (5).

The 21-item Depression Anxiety Stress Scale (DASS-21) and The Insomnia Severity Index (ISI) were applied to evaluate depression, anxiety and stress-related symptoms, and insomnia. To evaluate the potential effect of pandemic, participants were asked to rate these two scales twice in the same questionnaire, thinking on the last months during the pandemic and February 2020, which was just before the pandemic in Turkey.

DASS-21 is a 21-item, 4-point (0-3) Likert scale which is a revised, simplified version of the original DASS-42 developed by Lovibond [7,8]. ISI is a 7-item questionnaire with a five-point (0-4) answer scale and a total score ranging from 0 to 28 obtained by summing all items [9]. Higher scores are related to a more severe degree of symptoms for both scales. The validated Turkish versions of DASS-21 and ISI have been administered [10,11]. Scores greater than or equal to 10 for depression, 8 for anxiety, 15 for stress and 8 for insomnia were accepted as abnormal [8,9]. 

Statistical Analysis

Statistical analysis was performed using SPSS version 25.0 statistical software (IBM, Armonk, NY). Quantitative variables were described by the mean and standard deviation, while the frequency distribution described qualitative variables. Statistical difference between the DASS-21 and ISI scores before and after the pandemic was determined with Wilcoxon Signed Ranks Test. In univariate analysis, while independent samples t-test was used for variables with normal distribution, Mann-Whitney U test and Kruskal-Wallis test (Dunn test for paired comparisons) was used for non-normally distributed variables. Spearman's Rho correlation coefficient determined the correlation between quantitative variables. Multiple linear regression to determine factors affecting the DASS-21 subscale and ISI scores was performed. The level of statistical significance was set at <0.05.

## Results

Demographic characteristics

Among the respondents, 176 (48.9%) were female, and 258 (71.7%) were married. The mean age was 38.6±10.2 years (Range, 25 - 65 years). One hundred eighty-six (51.7%) were living in metropolitan areas where most COVID cases were accumulated during the first wave of the pandemic. Of the entire study population, 209 (58.1%) had one or more children. While 62 participants (17.2%) had a known chronic illness, 80 participants (22.2%) had one or more relatives with chronic diseases in their household. Eighty residents (22.2%), 220 fellows (61.1%), and 60 consultants (16.7%) composed the study population, and 54.4% of them were working in a tertiary hospital. While 115 participants (31.9%) have worked as ophthalmologists for less than five years, 75 of them (20.8%) had 20 years or more of experience in ophthalmology. Among the entire study group, 12 respondents (3.3%) were confirmed to be infected with COVID-19.

Working conditions in pandemic hospitals

**Figure 1:**
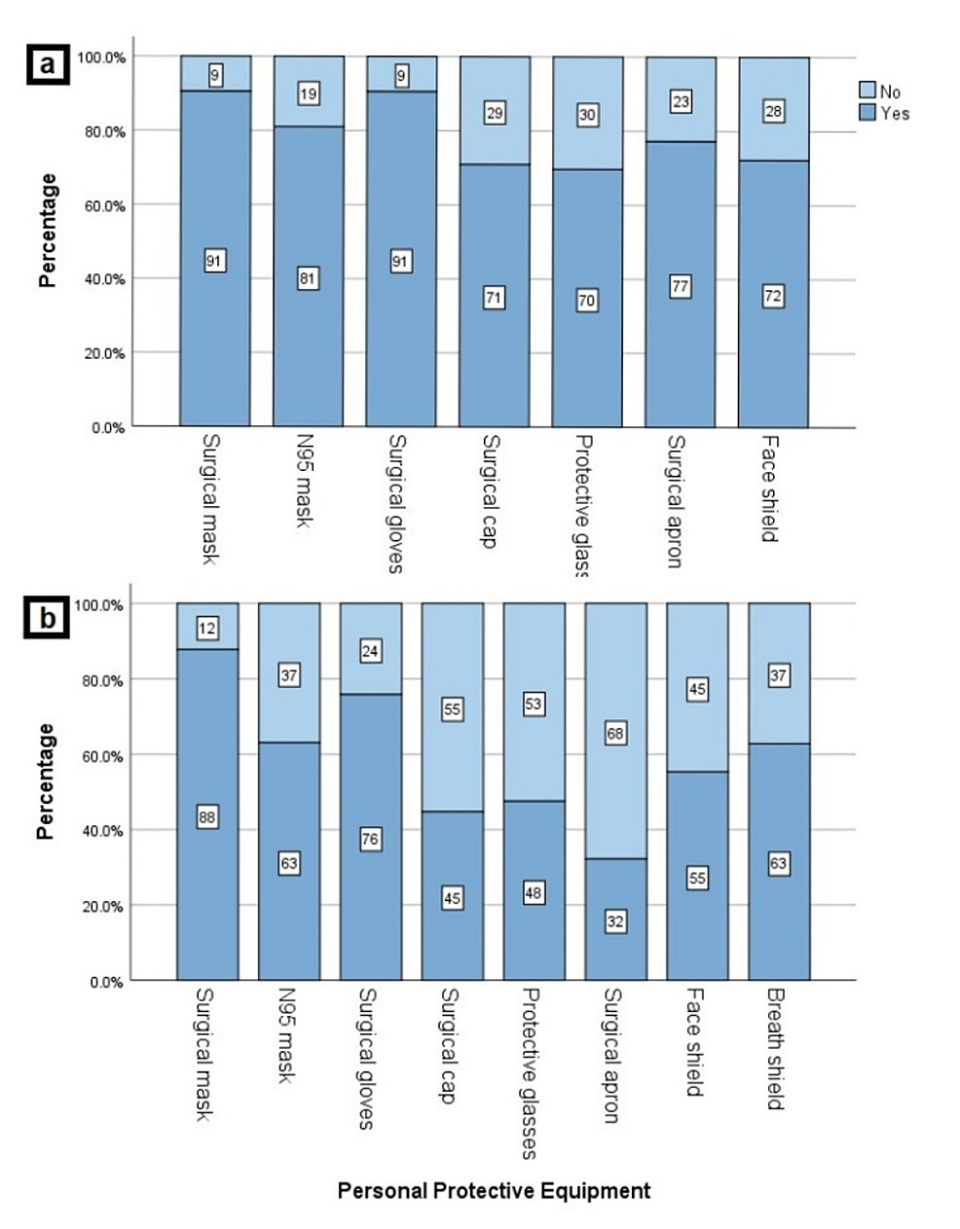
Extent of personal protective equipment provided by the hospital in sufficient quantity in (a) COVID and (b) Ophthalmology outpatient clinic and/or inpatient service

**Figure 2:**
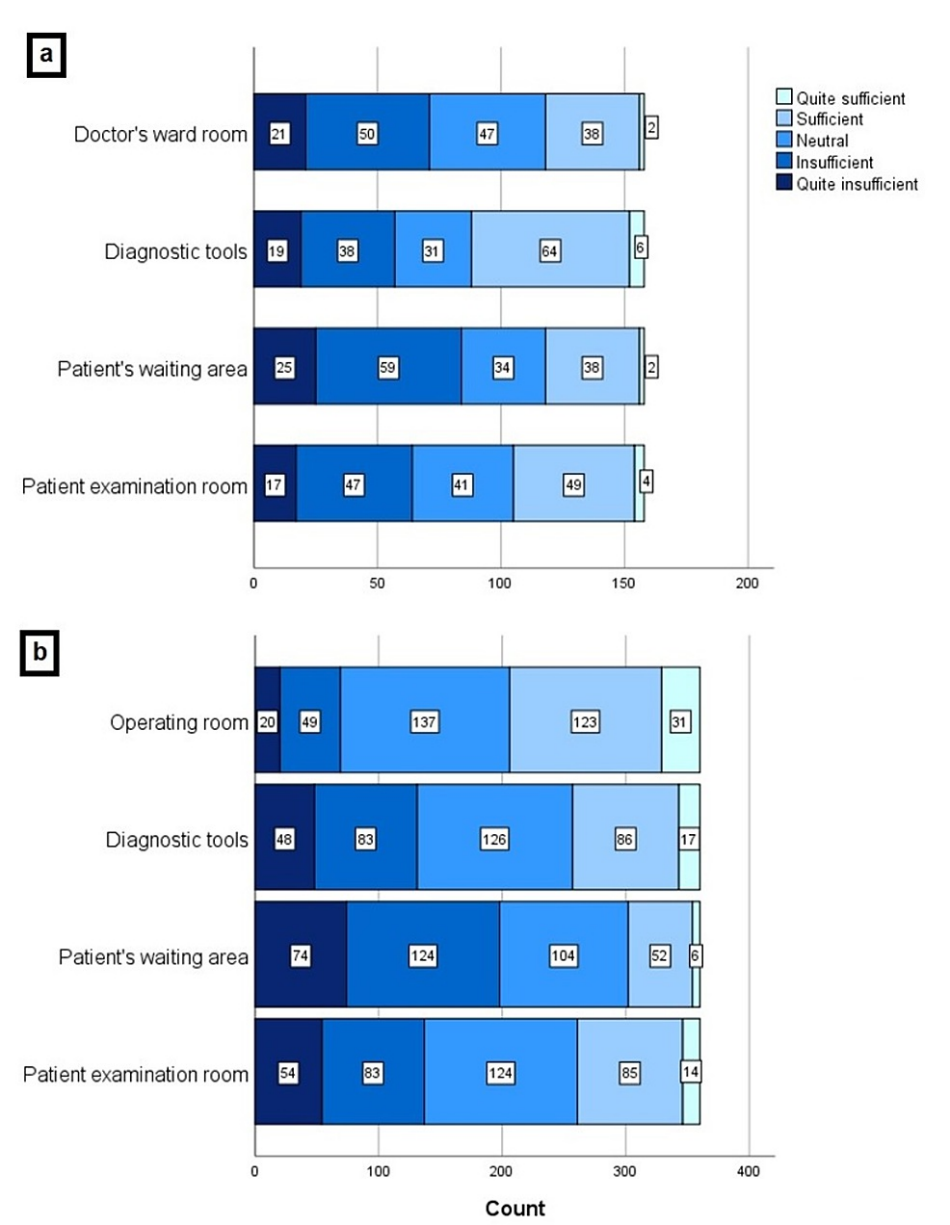
The satisfaction levels of physicians in terms of hygiene conditions in (a) COVID and (b) Ophthalmology outpatient clinic and/or inpatient service

Among our study population, 230 ophthalmologists (63.9%) were working in a pandemic hospital, and 158 participants (43.9%) were actively involved in COVID departments (frontline workers). Of 230 respondents, 184 (80.0%), 172 (74.8%), 153 (66.5%), 139 (60.4%), and 124 (53.9%) ophthalmologists declared that they were trained about the COVID-19 diagnosis-treatment schemes, the use of PPE, regulation of hospital environment, course of COVID-19 disease and elective/emergent ophthalmic procedures, respectively. However, 24 responders (10.4%) stated that the institution provided no training. Of 158 frontline workers who were actively involved in the diagnosis and treatment process of COVID-19 cases, 56 (35.4%) physicians were examining 0-10 patients per day, while 28 (17.7%) were examining 50 patients and above daily. The number of NPS taken daily by most of the physicians (n:99, 62.7%) was between 0-10. Among 158 frontline workers, 56 (35.4%) declared that they had all PPE in sufficient quantity in their COVID department. The extent of PPE in the COVID department is given in Figure 1a. As for satisfaction of hygiene in the COVID department, only one physician gave five points to all items, while eight physicians gave 1 point to all. Figure 2a shows the distribution of physicians according to their satisfaction levels of hygiene.

Working conditions in ophthalmology clinics

**Figure 3:**
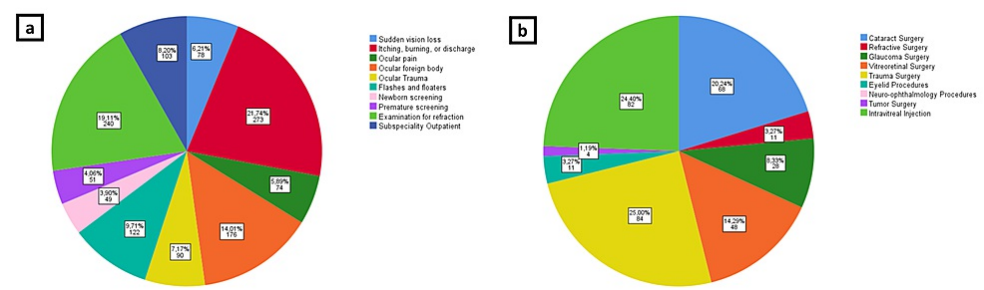
Distribution of frequent causes of ophthalmology clinic referrals (a) and ocular interventions perfomed by ophthalmologists (b) during the first wave of pandemic

During pandemic conditions, 115 ophthalmologists (31.9%) were seeing 10-25 patients, and 114 ophthalmologists (31.7%) have continued to examine 25 - 50 patients despite the restrictions. Despite having some of the important PPE in ophthalmology clinics, only 37 physicians (10.2%) reported having the entire PPE insufficient. The extent of PPE in ophthalmology clinics and the satisfaction levels of physicians in terms of hygiene conditions are given in Figure 1b and Figure 2b, respectively. The most common presenting symptom of patients was ocular "itching, burning, or discharge" followed by "examination for refraction", which were indicated by 294 (76.2%) and 261 (67.6%) of all participants, respectively. While 192 physicians (53.3%) declared that they did not perform any surgeries during the pandemic, "trauma surgery" (23.1%) and "intravitreal injection" (22.3%) were found to be the most commonly performed ocular interventions. The frequencies of presenting symptoms and ocular surgeries were given in Figure 3a and Figure 3b, respectively.

Psychological impact and associated factors

**Figure 4:**
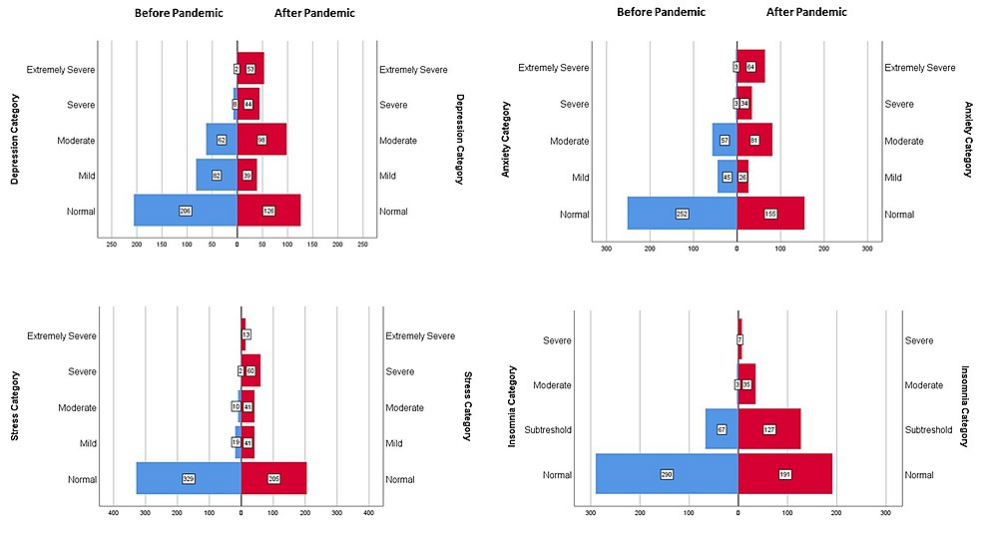
Change in number of participants in the severity groups of DASS-21* and ISI** before and during the first wave of pandemic (n) *DASS-21: Depression Anxiety Stress Scale -21(8) Recommended cut-off scores for severity labels (normal, mild, moderate, severe) are as follows: • Depression: 0-9 (Normal); 10-13 (Mild); 14-20 (Moderate); 21-27 (Severe); ≥28 (Extremely severe); • Anxiety: 0-7 (Normal); 8-9 (Mild); 10-14 (Moderate); 15-19 (Severe); ≥20 (Extremely severe); and • Stress: 0-14 (Normal); 15-18 (Mild); 19-25 (Moderate); 26-33 (Severe); ≥37 (Extremely severe). **ISI: Insomnia Severity Index (9) Recommended cut-off scores for severity labels are as follows: Normal (0–7), Subthreshold (8–14), Moderate (15–21), and Severe (22–28).

Table 1 presents the mean scores of depression, anxiety, stress and insomnia levels. Figure 4 shows the number of participants in the severity groups in the pandemic period compared to the pre-pandemic period. In the pandemic period, 234 (65.0%), 205 (56.9%), 155 (43.0%), and 169 (46.9%) ophthalmologists had depression, anxiety, stress and insomnia-related symptoms over the cut-off levels, respectively and levels of these symptoms were significantly higher during the pandemic compared to the pre-pandemic period (p<0.001 for all). Cronbach's alphas were found to be 0.856 for anxiety, 0.909 for depression and 0.915 for stress subscales in our study.

**Table 1:** Differences in mental health assessment scores between pre-pandemic and during pandemic for all participants DASS-21, Depression Anxiety Stress Scale

		Mean	Std Deviation	Std Error Mean	Significance (2-tail)
DASS-21 Depression	Pre-pandemic	7.49	6.24	0.329	<0.001*
	Pandemic	14.79	10.42	0.549	
DASS-21 Anxiety	Pre-pandemic	4.71	4.49	0.237	<0.001*
	Pandemic	10.59	8.61	0.454	
DASS-21 Stress	Pre-pandemic	8.52	5.87	0.310	<0.001*
	Pandemic	15.12	9.84	0.519	
Insomnia Severity Index	Pre-pandemic	4.48	3.61	0.191	<0.001*
	Pandemic	7.75	5.38	0.284	

Multiple comparisons revealed higher DASS-21 subscale and insomnia scores in female responders (p<0.001 for depression, anxiety and stress and p=0.002 for insomnia) and in those with a chronic disease (p<0.001, p=0.001, p=0.003, and p=0.004, respectively). There was a significant difference in stress levels in different age (p=0.049) and institution (p=0.046) groups, insomnia levels in different institution (p=0.023) groups and depression levels in different job titles (p=0.008). However, only the level of depression seen in residents compared to consultants remained significant in paired comparisons (p=0.006). The number of patients applied to COVID and ophthalmology clinics and the number of NPS taken in the COVID clinic per day did not significantly affect the MHS of participants. Supplementary Table summarized MHS scores influenced by demographic characteristics, job experience and workplace.

Ophthalmologists reported that the more they were trained about post-COVID regulations, the less anxiety they experienced (p=0.028). Higher levels of satisfaction with the hygiene conditions in COVID and the ophthalmology clinics lower the DASS-21 subscale and ISI scores (p=0.002 and <0.001 for depression; p=0.013 and <0.001 for stress; p=0.028 and p=0.004 for insomnia, and p=0.020 for anxiety only for ophthalmology clinic). Correlations between questionnaire scores and age, training status, adequacy of PPE and level of satisfaction with hygiene were given in Table 2.

**Table 2:** Correlations of between DASS-21 and ISI scores and age, training status, sufficiency of personal protective equipment and hygene satisfaction levels ANX, DASS-21 Anxiety score; COV, COVID clinic; DASS-21, Depression Anxiety Stress Scale; DEP, DASS-21 Depression score; INFO, Information status by institution; ISI, Insomnia severity index; OPH, Ophthalmology clinic; PPE, personal protective equipment; STR, DASS-21 Stress score * Correlation is significant at the 0.05 level (2-tailed) ** Correlation is significant at the 0.01 level (2-tailed)

Age	r	1									
	p										
Info	r	0.193** 0.003	1								
	p										
PPE (COV)	r	0.041 0.608	0.155 0.052	1							
	p										
Hygiene (COV)	r	0.242** 0.002	0.231** 0.003	0.210** 0.008	1						
	p										
PPE (OPH)	r	0.090 0.090	0.226** 0.001	0.354** <0.001	0.216** 0.006	1					
	p										
Hygene (OPH)	r	0.300** <0.001	0.261** <0.001	0.107 0.182	0.731** <0.001	0.257** <0.001	1				
	p										
DEP	r	0.024 0.647	-0.115 0.082	-0.004 0.959	0.245** 0.002	0.006 0.904	0.195** <0.001	1			
	p										
ANX	r	0.071 0.179	-0.145* 0.028	-0.025 0.754	-0.147 0.066	-0.001 0.990	-0.123* 0.020	0.784** <0.001	1		
	p										
STR	r	0.071 0.176	-0.128 0.053	-0.074 0.356	-0.198* 0.013	0.038 0.477	0.186** <0.001	0.831** <0.001	0.826** <0.001	1	
	p										
ISI	r	-0.017 0.742	-0.001 0.985	0.032 0.694	-0.175* 0.028	-0.032 0.551	0.150** 0.004	0.537** <0.001	0.582** <0.001	0.593** <0.001	1
	p										
		Age	Info	PPE (COV)	Hygiene (COV)	PPE (OPH)	Hyigene (OPH)	DEP	ANX	STR	ISI

Table 3 shows the multivariate predictors of higher DASS-21 and ISI scores. Multivariate regression analysis revealed that female gender, older age and lower hygiene satisfaction level in COVID-19 clinics were found independent predictors of high depression, anxiety and stress scores. Being a resident was a major predictor of depression.

**Table 3:** Multivariate predictors of higher DASS-21 subscale and ISI scores CI, Confidence interval; DASS-21, Depression Anxiety Stress Scale; ISI, Insomnia Severity Index; OR, Odds ratio

Variables	OR (95% CI)	p
Models for Depression		
Gender (female vs male)	5.07 (1.91 - 8.23)	0.002
Age (older vs younger)	0.26 (0.07 - 0.45)	0.006
Job title (Resident vs others)	7.77 (1.83 - 13.70)	0.011
Satisfaction level about hygiene in COVID clinic	-0.70 (-1.14 - -0.26)	0.002
Models for Anxiety		
Gender (female vs male)	3.87 (1.20 - 6.54)	0.005
Age (older vs younger)	0.17 (0.03 - 0.31)	0.018
Satisfaction level about hygiene in COVID clinic	-0.48 (-0.85 - -0.11)	0.01
Models for Stress		
Gender (female vs male)	4.67 (1.65 - 7.68)	0.003
Age (older vs younger)	0.22 (0.04 - 0.40)	0.016
Satisfaction level about hygiene in COVID clinic	-0.58 (-1.00 – -0.16)	0.006
Models for Insomnia		
Satisfaction level about hygiene in COVID clinic	-0.31 (-0.54 – -0.08)	0.008

**Figure 5:**
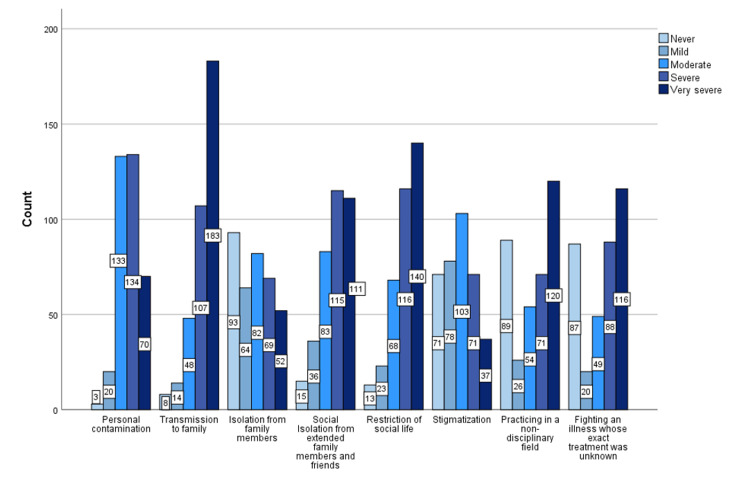
The factors that increased the distress of participants during the first wave of pandemic

The risks of "transmission to family members" and "restriction of social life", which were checked by 290 (80,5%) and 256 (71,1%) participants, respectively, were found to be the most common issues that caused "severe" and "very severe" distress in our study population. The factors that increased the distress of participants were broadly given in Figure 5.

## Discussion

The early periods of infectious disease outbreaks are when the adaptability and preparedness of the healthcare system can be best evaluated [12]. During this early period, healthcare practices and policies that are not fully structured yet and, as expected, the disease itself may cause higher physical and psychological vulnerability among HCW [5]. Therefore, in this study, we investigated ophthalmologists' practice patterns and mental health problems during the first wave of the pandemic. We found that 65% of participants had symptoms of depression, 56,9% had anxiety, 43% had stress, and 46,9% had insomnia. When compared to the pre-pandemic period, all these symptoms were significantly increased during the pandemic.

Among all medical disciplines, the most marked decrease in patient referrals has been reported in ophthalmology clinics during the COVID-19 pandemic. A recent study demonstrated that of 161 Turkish ophthalmologists who participated, 54% reported decreased weekly working hours, 53% were continuing routine outpatient clinics, and 67% performed only emergent surgeries [13]. While this may be an advantage for lowering the risk of infection, ophthalmologists and ENT doctors were reported to have the highest mortality amidst all HCW infected with COVID-19 during the initial outbreak in Wuhan, China [14]. Despite restrictions in the number of outpatients, most of the study population (63.6%) reported being in contact with up to 50 ophthalmology patients a day, who generally presented with possibly non-urgent symptoms of itching and burning in the eye and refraction examination. Breazzano et al. [15] reported that ophthalmology has the highest proportion of residents with confirmed COVID-19 infection in the New York area after frontline specialities. In this study, 12 participants (two residents, seven fellows and three consultants) reported that they were infected with COVID-19 during the first wave pandemic.

There is a controversy about which mask to wear during an ophthalmological examination. American Academy of Ophthalmology recommends the use of N95 masks in routine examinations [16]. On the other hand, MOH in Turkey and The Royal College of Ophthalmologists in the UK recommends N95 mask use only during aerosol-generating procedures [14,17]. Lin et al. [18] recommended using a surgical mask, gown, surgical cap, glasses, gloves and breath shield during a routine ophthalmological examination. Two studies among nurses indicated that those lacking access to an adequate amount of PPE were more likely to report symptoms of distress [19,20]. In the present study, the sufficiency of all PPE in the COVID clinic was above 70%.

On the other hand, the amount of specific PPE was insufficient in the ophthalmology clinic. Unlike previous studies, the sufficiency of PPE in both COVID and ophthalmology departments did not affect the MHS of participants in our study. We may hypothesize that this may be due to PPE is either provided in sufficient quantity by the institution or the physicians can purchase PPE with their budget.

Regular disinfection of the hospital environment is essential for ophthalmologists who encounter dozens of asymptomatic cases every day. Aytogan et al. [21] investigated the presence of SARS-CoV in several surfaces of an ophthalmology examination room after a routine clinic day and found samples from the face shield and the phoropter were positive for SARS-CoV. Regardless of the number of patients examined in COVID and ophthalmology clinics daily, the participants' satisfaction with the hygiene conditions of the ophthalmology clinic (in univariate analysis) and COVID clinic (in univariate and multivariate analysis) were negatively correlated with higher DASS-21 subscale and ISI scores.

The prevalence of depression, anxiety, stress and insomnia in this study were higher than other studies conducted with healthcare workers in other countries [4,22]. However, they were lower than the findings of the study conducted in Turkey, which reported the prevalence of 77.6%, 60.2%, 50.4%, and 76.4% for depression, anxiety, distress and insomnia, respectively [23]. Different time frames and designs of these studies may explain the different prevalence rates found. In our study, the female gender consistently showed a higher prevalence of psychological distress across all scales, in agreement with previous studies among HCW [3,4].

Please keep the original version of this sentence for a better understanding of what I mean: “Participants either having a chronic illness or having a relative with chronic illness in their families were found to experience more anxiety which can be explained by a more severe course of the disease in patients with a chronic illness''. Similar to Khanna et al. [24], we found a higher prevalence of depression in residents. Factors such as being young and inexperienced physicians, higher workload and interruption of their educational process can explain this situation. Married participants had lower DASS-21 subscale scores compared to singles. Dyadic coping and social support may be the possible advantages of married participants [25].

ISI scores of those working in the pandemic hospitals were higher, possibly due to participants being on-call for 24 hours in these hospitals. Although participants diagnosed with COVID-19 had higher scores in all MHS scales, the difference was not significant, which might result from the small sample size of COVID-positive ones.

Today, a better understanding of the COVID-19 diagnosis and treatment process, social immunization secondary contracting the disease or vaccination, and establishing constructed practices in health care facilities has enabled HCW to work in more comfortable conditions physically and psychologically compared to the beginning of the pandemic. However, each new attack caused by the mutant variants of the virus causes the whole process to start over, indicating that COVID-19 will be in our lives for a long time.

There are some limitations of this study. Retrospective questions about mental health before pandemic may cause recall bias. Besides, the survey was conducted within three months, and there is a lack of longitudinal follow up. Finally, although the scales may give some diagnostic clues, the actual diagnosis of mood and anxiety disorders can only be made after a clinician's examination.

## Conclusions

During the first wave of the COVID-19 outbreak, ophthalmologists have suffered from physical exhaustion and a psychological burden. They need health protection, improved working conditions such as sufficient PPE, better environmental sanitation, work hours to provide adequate rest, and rehabilitation programs to strengthen their resilience. Precautions to support the education of residents must also be taken. The symptoms of depression, anxiety, stress and insomnia can be prevented from turning into chronic problems with appropriate mental health management.

## Appendices

**Table 4:** Supplementary Table. Mental health status scores by demographic characteristics and working conditions during the first wave of the pandemic Test statistics: t value, for parametric tests; z value and χ2 value for non-parametric tests

Characteristics	N (%)	Depression			Anxiety		Stress		Insomnia	
		Mean ± SD	Median (min-max)		Mean ± SD	Median (min-max)	Mean ± SD	Median (min-max)	Mean ± SD	Median (min-max)
Sociodemographical Characteristics (n=360)										
Gender										
Female	176 (48.9%)	17.13 ± 10.27	16.0 (0-42)		12.51 ± 8.27	11.0 (0-32)	17.35 ± 9.39	16.0 (0-42)	8.63 ± 5.19	9 (0-24)
Male	184 (51.1%)	12.57 ± 10.10	12.0 (0-42)		8.76 ± 8.54	6.0 (0-42)	12.98 ± 9.81	12.0 (0-42)	6.91 ± 5.45	6 (0-25)
t		4.24			4.22		4.31		3.04	
p value		<0.001*			<0.001*		<0.001*		0.002	
Age range										
25-35	163 (45.3%)	14.34 ± 10.20		14.0 (0–42)	10.11 ± 8.48	8.0 (0–32)	14.42 ± 10.01	12.0 (0–42)	7.91 ± 5.40	7.0 (0–24)
36-45	113 (31.4%)	14.58 ± 10.62		14.0 (0–40)	10.78 ± 8.90	10.0 (0–40)	15.22 ± 9.84	14.0 (0–42)	7.60 ± 5.13	7.0 (0–24)
46-55	54 (15.0%)	17.70 ± 9.90		16.0 (0–40)	12.11 ± 8.34	12.0 (0–34)	18.04 ± 8.65	16.0 (2–40)	8.59 ± 5.88	8.5 (0–25)
56 and above	30 (8.3%)	12.80 ± 11.28		13.0 (0–42)	9.80 ± 8.70	9.0 (0–42)	13.27 ± 10.29	13 (0–40)	5.93 ± 5.09	6.0 (0–21)
χ^2^		6.776			3.319		7.878		4.686	
p value		0.079			0.345		0.049*		0.182	
Marital status										
Single	102 (28.3%)	17.61 ± 10.46	17.0 (0-42)		12.59 ± 9.84	11.0 (0-42)	17.33 ± 9.96	14 .0 (0-42)	8.58 ± 5.71	8.0 (0-23)
Married	258 (71.7%)	13.68 ± 10.21	14.0 (0-42)		9.81 ± 7.95	8.0 (0-34)	14.24 ± 9.68	14.0 (0-42)	7.42 ± 5.23	7.0 (0-25)
t		3.26			2.78		2.70		1.84	
p value		0.001*			0.006*		0.007*		0.067	
Parental status										
No children	151 (41.9%)	15.89 ± 10.37	16.0 (0-42)		10.98 ± 9.14	8.0 (0-42)	15.70 ± 9.74	14.0 (0-42)	8.21 ± 5.41	8.0 (0-23)
Has children	209 (58.1%)	14.00 ± 10.41	14.0 (0-42)		10.32 ± 8.21	8.0 (0-34)	14.70 ± 9.92	14.0 (0-42)	7.42 ± 5.35	7.0 (0-25)
t		1.70			0.72		0.94		1.36	
p value		0.08			0.47		0.34		0.17	
Has chronic illness										
No	298 (82.8%)	13.87 ± 9.90	14.0 (0-42)		9.93 ± 8.36	8.0 (0-40)	14.42 ± 5.59	14.0 (0-42)	7.38 ± 5.19	7.0 (0-24)
Yes	62 (17.2%)	19.23 ± 11.75	19.0 (0-42)		13.81 ± 9.10	12.0 (0-42)	18.48 ± 10.41	16.0 (0-40)	9.52 ± 5.96	8.0 (0-25)
t		-3.74			-3.27		-2.99		-2.86	
p value		<0.001*			0.001*		0.003*		0.004*	
Household with chronic illness										
No	280 (77.8%)	14.49 ± 10.27	14.0 (0-42)		10.11 ± 8.30	8.0 (0-40)	14.80 ± 9.63	14.0 (0-42)	7.34 ± 5.17	7.0 (0-24)
Yes	80 (22.2%)	15.88 ± 10.93	16.0 (0-42)		12.30 ± 9.45	10.0 (0-42)	16.23 ± 10.55	14.0 (0-40)	9.20 ± 5.87	9.0 (0-25)
t		-1.05			-2.01		-1.14		-2.75	
p value		0.29			0.04*		0.25		0.006*	
Diagnosed with COVID-19										
No	348 (96.7%)	14.72 ± 10.44		14.0 (0–42)	10.45 ± 8.54	8.0 (0-42)	14.95 ± 9.84	14.0 (0 -42)	7.74 ± 5.37	7.0 (0 – 21)
Yes	12 (3.3%)	17.00 ± 9.89		17.0 (0–32)	14.67 ± 9.81	16.0 (0–30)	20.0 ± 9.06	23.0 (0–30)	8.00 ± 6.09	9.0 (0–17)
z		0.844			1.555		1.912		0.187	
p value		0.399			0.120		0.056		0.852	
Working Conditions (n=360)										
Pandemic Hospital										
No	130 (%36.1)	14.52 ± 10.25	14.0 (0-38)		10.43 ± 8.30	9.0 (0-40)	14.51 ± 9.16	14.0 (0-42)	6.82 ± 4.93	6.5 (0-25)
Yes	230 (%63.9)	14.95 ± 10.53	14.0 (0-42)		10.69 ± 8.79	8.0 (0-42)	15.46 ± 10.21	14.0 (0-42)	8.27 ± 5.56	8.0 (0-24)
t		-0.37			-0.27		-0.88		-2.47	
p value		0.71			0.78		0.37		0.014*	
Frontline										
No	202 (56.1%)	14.48 ± 10.12	14.0 (0-40)		10.21 ± 8.18	8.0 (0-40)	14.58 ± 9.72	14.0 (0–42)	7.29 ± 5.29	7.0 (0-25)
Yes	158 (43.9%)	15.20 ± 10.81	14.0 (0-42)		11.09 ± 9.13	9.0 (0-42)	15.80 ± 9.98	14.0 (0–42)	8.34 ± 5.46	8.0 (0-42)
t		-0.65			-0.96		-1.16		-1.82	
p value		0.51			0.33		0.24		0.06	
Job Title										
Resident	80 (22.2%)	16.78 ± 9.92	16.0 (0–40)		11.08 ± 9.02	8.0 (0–32)	16.20 ± 10.03	14.0 (0–40)	8.38 ± 5.55	8.5 (0–22)
Fellow	220 (61.1%)	15.01 ± 10.85	14.0 (0–42)		10.85 ± 8.91	9.0 (0–42)	15.22 ± 9.81	14.0 (0–42)	7.50 ± 5.22	7.0 (0–25)
Consultant	60 (16.7%)	11.37 ± 8.65	11.0 (0–36)		9.03 ± 6.63	10.0 (0–24)	13.30 ± 9.62	14.0 (0–40)	7.82 ± 5.77	8.0 (0–23)
χ^2^		9.740			0.992		2.296		1.516	
p value		0.008*			0.609		0.317		0.469	
Practice Time										
0-5 years	115 (31.9%)	15.18 ± 9.96	14.0 (0–40)		10.19 ± 8.64	8.0 (0–32)	14.97 ± 9.58	14.0 (0–40)	7.78 ± 5.36	7.0 (0–22)
6-10 years	75 (20.8%)	15.41 ± 11.64	14.0 (0–42)		11.76 ± 9.72	10.0 (0–40)	16.00 ± 11.03	14.0 (0–42)	8.12 ± 5.34	7.0 (0–24)
10-20 years	95 (26.4%)	13.43 ± 9.94	14.0 (0–38)		9.41 ± 7.89	8.0 (0–30)	13.58 ± 9.50	14.0 (0–34)	7.83 ± 5.70	7.0 (0–25)
21 years or above	75 (20.8%)	15.32 ± 10.47	14.0 (0–42)		11.55 ± 8.14	12.0 (0–42)	16.40 ± 9.32	16.0 (0–40)	7.23 ± 5.12	7.0 (0–23)
χ^2^		1.826			4.527		3.848		1.037	
p value		0.609			0.210		0.278		0.792	
Institution										
State Hospital	87 (24.2%)	16.30 ± 10.69	14.0 (0–42)		12.53 ± 9.60	10.0 (0–42)	17.56 ± 9.62	14.0 (0–42)	8.86 ± 5.50	8.0 (1–25)
Private Practice	32 (8.9%)	14.73 ± 14.00	14.0 (0–40)		9.32 ± 7.85	8.0 (0–28)	12.98 ± 9.02	12.0 (0–30)	6.15 ± 4.48	7.0 (0–14)
University Hospital	196 (54.4%)	14.29 ± 10.22	14.0 (0–42)		10.06 ± 8.22	8.0 (0–34)	14.73 ± 9.96	14.0 (0–42)	7.84 ± 5.39	7.0 (0–23)
Branch Hospital	36 (10.0%)	14.00 ± 10.89	14.0 (0–38)		10.28 ± 8.59	9.0 (0–28)	13.72 ± 9.86	11.0 (0–32)	6.42 ± 5.49	5.0 (0–24)
χ^2^		2.261			4.801		7.984		9.554	
p value		0.520			0.187		0.046*		0.023*	
COVID Department (n=158)										
Number of patients applied per day										
0-10	56 (35.4%)	16.64 ± 12.21		16.0 (0–42)	12.25 ± 9.70	10.0 (0–42)	16.89 ± 11.02	16.0 (0–42)	8.84 ± 5.67	8.0 (0–21)
10-25	47 (29.7%)	14.51 ± 9.55		14.0 (0–36)	10.85 ± 9.43	8.0 (0–32)	14.94 ± 9.92	14.0 (0–38)	8.55 ± 5.39	8.0 (0–22)
25-50	27 (17.1%)	13.56 ± 11.35		12.0 (0–40)	10.52 ± 8.22	10.0 (0–34)	13.70 ± 9.39	12.0 (0–34)	7.33 ± 5.07	6.0 (0–22)
50 and above	28 (17.7%)	15.07 ± 9.43		14.0 (0–38)	9.71 ± 8.44	8.0 (0–26)	17.07 ± 8.35	14.0 (0–40)	7.93 ± 5.61	6.0 (1–24)
χ^2^		1.317			1.872		2.825		1.833	
p value		0.725			0.599		0.419		0.608	
Number of nasopharyngeal aspiration samples taken										
0-5	99 (62.7%)	16.30 ± 11.41		16.0 (0–42)	11.98 ± 9.53	10.0 (0–42)	15.39 ± 10.63	14.0 (0–40)	8.68 ± 5.90	8.0 (0–24)
5-15	36 (22.8%)	12.61 ± 10.47		12.0 (0–42)	10.50 ± 9.56	8.0 (0–32)	17.05 ± 9.94	12.0 (0–42)	7.78 ± 4.33	8.0 (0–16)
15 and above	23 (14.6%)	14.52 ± 7.95		14.0 (2–40)	8.17 ± 5.62	8.0 (0–20)	15.57 ± 6.90	14.0 (4–32)	7.74 ± 5.13	6.0 (1–21)
χ^2^		3.238			2.596		0.389		0.604	
p value		0.198			0.273		0.823		0.739	
Ophthalmology Department (n=360)										
Number of patients applied per day										
0-10	71 (19.7%)	18.67 ± 14.27		18.0 (0–42)	14.33 ± 12.22	10.0 (0–42)	17.83 ± 12.12	19.0 (2–40)	9.92 ± 6.01	10.5 (0–21)
10-25	115 (31.9%)	14.56 ± 9.53		14.0 (0–36)	9.21 ± 7.54	8.0 (0–32)	13.91 ± 8.95	12.0 (0–38)	7.02 ± 4.98	6.0 (0–22)
25-50	114 (31.7%)	15.08 ± 11.30		14.0 (0–42)	12.20 ± 9.11	10.0 (0–34)	16.52 ± 10.0	15.0 (0–42)	8.08 ± 5.29	8.0 (0–22)
50 and above	60 (16.7%)	14.0 ± 9.0		14.0 (0–38)	9.80 ± 8.14	8.0 (0–26)	15.71 ± 9.66	14.0 (0–40)	9.10 ± 5.63	7.0 (0–24)
χ^2^		3.108			2.949		7.219		1.902	
p value		0.375			0.400		0.065		0.593	
